# Barriers and Enablers to Food Waste Recycling: A Mixed Methods Study amongst UK Citizens

**DOI:** 10.3390/ijerph19052729

**Published:** 2022-02-26

**Authors:** Ayşe Lisa Allison, Fabiana Lorencatto, Susan Michie, Mark Miodownik

**Affiliations:** 1 Plastic Waste Innovation Hub, University College London, London W1T 4TJ, UK; s.michie@ucl.ac.uk (S.M.); m.miodownik@ucl.ac.uk (M.M.); 2 Centre for Behaviour Change, University College London, London WC1E 7JE, UK; f.lorencatto@ucl.ac.uk

**Keywords:** behaviour change, Behaviour Change Wheel, circular economy, citizens, COM-B, food waste, intervention, policy, recycling

## Abstract

We aim to identify influences on UK citizens’ household food waste recycling as a basis for designing strategies to increase household food waste collection rates via local services. Using a UK dataset (*n* = 1801) and the COM-B (Capability–Opportunity–Motivation–Behaviour) model as a theoretical framework, we conduct quantitative regression and supporting thematic analyses to investigate influences on citizens’ recycling of food waste. Results show that automatic motivation (e.g., emotions and habit) and psychological capability (e.g., knowledge) predict household food waste recycling. Physical opportunity (i.e., dealing with food waste in other ways such as home-composting or feeding pets/strays, time and financial costs) was the main barrier to recycling food waste identified in thematic analyses. Participants also reported automatic motivation-related barriers such as concerns over pests, odour, hygiene and local authorities’ food waste collection capabilities. Based on findings we recommend the development of clear, consistent communications aimed at creating positive social norms relating to recycling and increasing knowledge of what can and cannot be put in food waste bins. Improved functional design and free distribution of bins and compostable caddy liners developed according to user-centred needs for cleanliness, convenience and hygiene are also needed. These will not be sufficient without a nationally uniform, efficient and reliable system of household food waste collection.

## 1. Introduction

### 1.1. General Introduction

Landfill and incineration are two of the least environmentally-friendly methods of processing food waste owing to excess production of potent greenhouse gases, e.g., carbon dioxide (CO_2_) and methane (CH_4_) [1,2,3,4]. It also represents a missed opportunity to either compost the food waste and thus create a valuable nutrient source for crops and improve soil health, or to anaerobically digest the food waste to create renewable energy in the form of biogas. Diverting food waste from incineration and landfill is, therefore, critical for sustainability efforts. Recognising that preventative strategies to minimise food waste (e.g., via source reduction, donation or conversion to animal feed) are the most beneficial in terms of environmental, societal and economic impact, recycling (i.e., via composting systems) is the next best option for dealing with food waste once it has entered the waste stream [5,6,7].

Successful food waste recycling, however, relies on high rates of food waste collection by local authorities [8]. This, in turn, relies on citizens correctly segregating their food waste for collection. Several different systems for collecting food waste were implemented across Europe. There are highly efficient systems based on source separation of food in Austria, Slovenia, Belgium and Germany where the bio-waste capture rate (This percentage represents food waste collected as a percentage of food waste generated) is over 60% [9]. The success of these systems is owed, in part, to technical factors, i.e., widescale implementation of a simple to use, nationally uniform, efficient and reliable waste collection service. It is also owed to high citizen engagement with food waste recycling schemes which was achieved through effective behaviour change interventions, including educational and motivational public campaigns to create positive social norms about household food recycling and the provision of free bins and compostable liners [10]. Citizen behaviour is therefore critical for initiating the end-of-life pathway of food waste, ensuring recycling takes place.

### 1.2. Literature Review

#### 1.2.1. The UK Food Waste Context

The UK generates 9.5 million tonnes of food waste annually, 70% of which comes from households [11]. To meet their sustainability targets, the government aims to have increased food waste capture by developing comprehensive nationwide food waste collection services by 2023 [12]. At the time of this study, in 2021, many households across the UK do not have access to separate food waste collection services, with low rates of food waste recycling compared to other European countries [13]. Food waste collection services are better in the UK’s devolved nations. In Wales, weekly food waste collections are offered to 99% of Welsh households [14,15]. However, in England, where local authorities make the decisions on collection and recycling operations, separate food waste collection services are available to fewer than half of all households [16]. England is responsible for over three quarters (82%) of UK biodegradable municipal waste (food waste, green waste, cardboard and paper) sent to landfills, generating 5.4 million tonnes of the 6.6 million tonnes UK total in 2019 [13].

In the UK, there are three different routes for processing food waste, two of which are commercial services, anaerobic digestion (AD) and In-Vessel Composting (IVC), and one of which is home/community composting. In IVC, organic waste is composted in temperature-controlled aerobic conditions (i.e., in the presence of oxygen) to create nutrient-rich compost. In AD, organic waste is broken down by microorganisms in an oxygen-free environment which yields biogas (principally methane) and a liquid digestate. For technical details of different composting methods, see [9,17,18]. Local home/community composting is an effective organic waste management option as it reduces demand for separate collection [19] thereby reducing the associated environmental and financial costs of waste transport and management [20]. This is particularly relevant in sparsely populated, rural, areas [9]. However, it may not be feasible for the majority of urban-dwelling UK citizens who live in densely populated housing often without access to a garden [21]. Local home/community is also an aerobic process of composting food waste, however, there is little explicit control of temperature, humidity, and security against vermin. It therefore may not be appropriate for processing some types of mainstream organic waste due to the controlled environments required for these materials to degrade safely or without attracting vermin (e.g., compostable plastic packaging, cooked vegetables, meat, dairy, skin and bones). Commercial composting, enabled by household food waste collection services, is therefore the most practicable policy option to ensure that food waste recycling is effective and safe for large and dense populations.

#### 1.2.2. Citizen Behaviour Change

**Figure 1:**
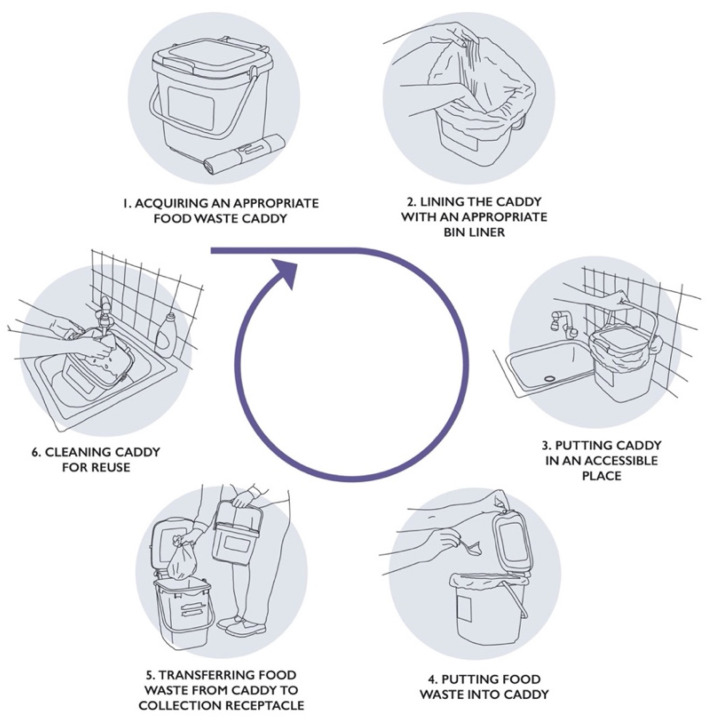
System of interacting food waste recycling behaviours.

The success of food waste collection schemes depends on citizens’ behaviour; for the UK, this will require population-wide adoption of a new set of household food waste recycling behaviours that are not currently part of most people’s routines. “Food waste recycling” requires interconnected behaviours enacted by citizens, including: 1. acquiring an appropriate food waste caddy, 2. lining the caddy with an appropriate bin liner, 3. putting it in an accessible place (e.g., on the kitchen counter or under the sink), 4. putting waste food into it, 5. transferring the food waste from container to the collection receptacle, and 6. cleaning the food waste caddy for reuse (see Figure 1).

As household food waste recycling is a relatively new behaviour in terms of an integrated UK waste management strategy, there is limited empirical research on this topic. To achieve the adoption of this new set of recycling behaviours, knowledge of influences on current and desired food waste recycling behaviour is needed. For instance, this includes understanding why those who currently have access to food waste recycling services do not recycle their food waste. It also requires investigating citizens’ use of compostable caddy liners since hygiene concerns and the perceived mess associated with handling food waste were identified as potential barriers to food waste recycling [22]. While some preliminary evidence suggests that compostable bags might be appealing to UK citizens by reducing the “ick” factor [22], little is known scientifically about current rates of use and what the potential barriers to their adoption might be. It is also increasingly acknowledged that “acceptability” should be considered when designing, evaluating and implementing interventions [23]. However, little is known about how prepared the UK public may feel about having to adopt a new set of recycling behaviours. Understanding UK citizens’ acceptance and readiness for nationwide food waste collection services can therefore increase the likely effectiveness of implementation efforts.

#### 1.2.3. Theoretical Framework

Guidance for developing and evaluating the kinds of “complex” interventions needed to achieve such behaviour change point to the importance of ensuring they are theoretically grounded and informed by evidence [24,25]. A first step in the process of developing interventions likely to be effective is to conduct a “behavioural analysis” of citizen food waste recycling. Such an analysis identifies the influences on behaviour (i.e., factors that prevent, maintain or promote recycling). This knowledge informs targets for interventions.

**Figure 2:**
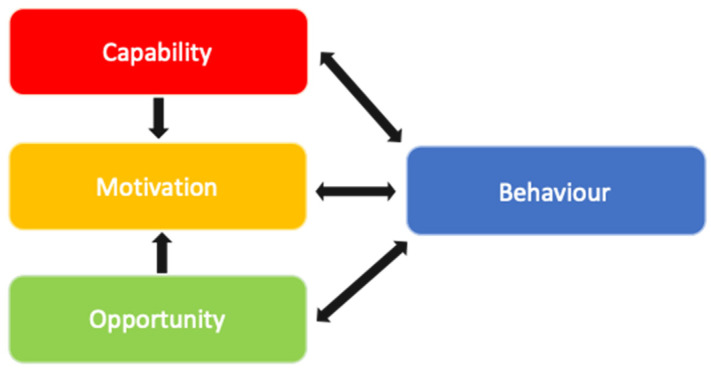
COM-B—a model for understanding behavioural influences [26,27] (Michie et al., 2011; Michie et al., 2014).

A framework that has been widely used to conduct such a behavioural analysis (i.e., identify behavioural targets for interventions) is the COM-B model (Capability–Opportunity–Motivation–Behaviour model; Figure 2) [26,27]. COM-B posits that for a behaviour to occur there must be the capability, opportunity and motivation to perform the behaviour. Capability can be psychological (e.g., knowledge) or physical (e.g., skills); opportunity can be social (e.g., social norms) or physical (e.g., environmental resources); motivation can be automatic (e.g., habits) or reflective (e.g., beliefs, intentions). Such influences can be barriers, preventing or hindering a target behaviour, or enablers that promote or maintain a target behaviour. COM-B was developed as part of a wider intervention development framework and methodology called the Behaviour Change Wheel (BCW). The BCW provides a structured approach to designing behaviour change interventions for individuals, organisations and populations. Using the BCW during intervention development provides a systematic and comprehensive analysis of available intervention options and their potential costs and benefits by using behaviour change theory and the available evidence. We refer readers elsewhere for more detail on the BCW [26,27].

**Figure 3:**
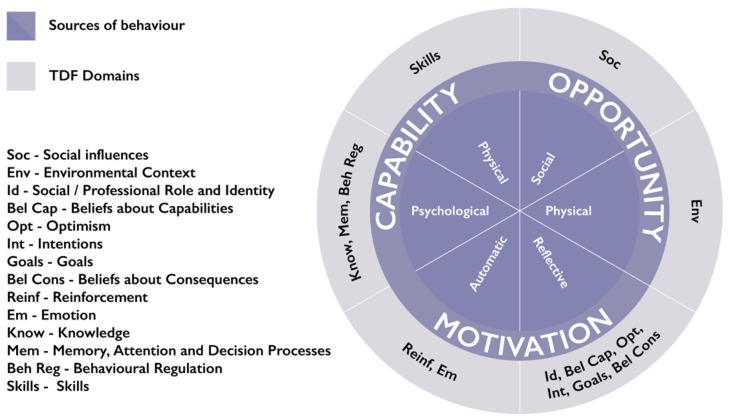
Relationship between TDF and COM-B components [26,27,28] (Cane et al., 2013; Michie et al. 2011; 2014).

A more granular, elaborated version of COM-B is the Theoretical Domains Framework (TDF; Appendix A) [28]. The TDF comprises 14 theoretical domains, representing the individual, socio-cultural and environmental factors influencing behaviour. These domains include people’s knowledge and skills, memory, attention and decision-making processes, beliefs about capabilities and consequences, goals and emotions as well as physical and social environmental factors. The relationship between COM-B components and TDF domains are shown in Figure 3. Both COM-B and TDF are used by researchers, policymakers and intervention designers to identify modifiable influences on a target behaviour to inform interventions and policies.

While these frameworks have been mostly applied in patient and healthcare professional populations [25,29,30,31,32,33,34], they have also been applied to understanding and changing a range of environmentally- and socially-significant target behaviours such as: recycling [35], water consumption [36], reusable face-mask use [37], plastic packaging consumption [38,39], fuel-stacking [40], higher-welfare food choice [41], data leakage in financial organisations [42] and participation in citizen science [43].

### 1.3. Study Aims

There is a strong rationale and great potential utility in applying behaviour change frameworks to expand our understanding of household food waste recycling as a basis for designing citizen behaviour change strategies to increase council food waste collection rates. There currently exists no empirical investigations using behaviour change theoretical frameworks to identify influences on household food waste recycling behaviour. Such evidence is required to provide the high-quality evidence needed to contribute to effective intervention development. In this study, we therefore conduct a mixed-methods study to identify barriers and enablers to recycling food waste amongst a sample of UK citizens. To this end, our specific research questions are:

What are the capability-, opportunity- and motivation-related influences on food waste recycling amongst UK citizens?

For UK citizens with access to council waste collection services, what reasons do they give for not recycling food waste?

For UK citizens with access to council waste collection services, what reasons do they give for not using compostable caddy liners?

What reasons do UK citizens give for feeling unprepared for nationwide food waste collection services?

## 2. Methods

### 2.1. Design

This was a mixed-methods cross-sectional survey study [44,45]. In line with prior rationales, we chose mixed-methods in order to achieve “triangulation” (i.e., seeking corroboration between quantitative and qualitative data to increase validity of findings) and “completeness” (i.e., combining research approaches to provide a more comprehensive picture of the study phenomenon) [46,47,48]. For regression analyses, predictor variables were components of the COM-B model; psychological capability, physical opportunity, social opportunity, automatic motivation and reflective motivation, and the outcome variable was food waste recycling behaviour; these are operationalised in Section 2.5. Thematic analysis was conducted on qualitative data as detailed in Section 2.5.

### 2.2. Participants and Recruitment

Study participants consisted of UK citizens, aged 18 and above. Participants were recruited via Prolific—a professional data collection service [49]—and opportunity sampling, i.e., advertising the study via: (a) social media (including Hertfordshire Council’s official Twitter page, the UCL Centre for Behaviour Change’s official Twitter account and the UCL Environment and Behaviour Hub’s professional LinkedIn group); (b) email (including the Big Compost Experiment’s [50] mailing lists and Hertfordshire Council’s mailing lists of residents). Participants recruited via Prolific were compensated for their time at a rate GBP 10.89/h. Prolific ensures a representative sample in terms of gender, age and ethnicity for UK participants. Participants recruited via email and social media took part voluntarily.

### 2.3. Questionnaire

A survey, hosted by Qualtrics [51] was developed in line with guidance for using COM-B and TDF in implementation research [39,52]. We developed a preliminary set of survey items from a prior survey investigating barriers and enablers to single-use and reusable cup use [39]. The survey went through two rounds of paper piloting: once through a team of behavioural science experts and second, through an interdisciplinary team of circular economy researchers with non-behavioural science expertise. The survey was then built online and went through another round of piloting with a different group of behavioural science experts. Minor changes were made to the ordering of items and wording to increase clarity. The relationship between our survey items, psychological constructs, COM-B categories and TDF domains are shown in Supplementary File S1. The final survey consisted of demographic questions, questions about current food waste recycling behaviour, a series of 5-point Likert scale items aimed at assessing potential capability, opportunity and motivation-related influences on food waste recycling behaviour and, questions on participants’ awareness of and readiness for the UK government’s plan to roll-out nation-wide food waste collection. The survey is openly available via Open Science Framework (OSF) at https://osf.io/d5jw7/ (accessed on 24 February 2022).

### 2.4. Procedure

Ethical approval was received from University College London (project ID: CEHP/2020/579, data protection: Z6364106/2020/02/86). Participants accessed the survey link via an online web link. The survey took approximately five minutes to complete. Informed consent was obtained prior to any data collection. Data collection occurred between 11 May 2021 and 21 June 2021.

### 2.5. Data Analysis

Statistical analyses were performed in SPSS (IBM, Armonk, NY, USA) [53]. Data were analysed in a phased approach. First, assumption checks were made. Then, prior to running the regression, we investigated the impact of demographic variables on the outcome variable. To run these tests, we ran correlations using Pearson’s r for continuous variables and independent t-tests and one-way ANOVAs to determine whether the differences between group means of categorical variables were statistically significant. The demographic variables that were significantly associated with our outcome variable were subsequently controlled for in the regression analyses.

Hierarchical multiple linear regression was performed to identify factors associated with recycling food waste. ANOVA was used to examine (using interval plot) and compare (using the Tukey method and 95% confidence) the group means and to determine how well the model fits the data (calculation of S, R2 and R2 pred.) and if the model meets the assumptions of the analysis (by interpreting the residuals versus fits plot and normality plot of the residuals).

The outcome variable in the regression analyses was food waste recycling behaviour operationalised as the frequency of food waste recycling (as measured by the survey item “How often do you use the food waste bin when disposing of food waste?”). The following responses were used: always = 5, most of the time = 4, about half the time = 3, sometimes = 2, never = 1. Participants who said no to using a separate food waste bin for recycling food waste were coded as “never = 1” and also entered into analysis.

The predictor variables were psychological capability, physical opportunity, social opportunity, reflective motivation and automatic motivation (components of the COM-B model). Responses to each item were coded so that 1 = disagree, 2 = somewhat disagree, 3 = not sure, 4 = somewhat agree, 5 = agree. Negatively worded items were reverse coded so that a score of 5 always indicated high capability, opportunity or motivation and 1 represented a lack of capability, opportunity or motivation. The mean COM-B scores were calculated for each participant. For example, if there were three items measuring social opportunity, the average score of those three items was taken as that person’s score for social opportunity. COM-B scales were considered to represent an acceptable level of internal consistency if the Cronbach’s alpha value fell from 0.5 to 0.7 and a good level of consistency if the Cronbach’s alpha value was more than 0.7 [54,55,56,57]. Cronbach’s alpha (α) tests to see if multiple-question Likert scale surveys are reliable. It measures reliability via measuring the internal consistency of survey items. Survey items measure “latent variables”—hidden or unobservable variables such as “psychological capability”. Cronbach’s alpha will tell you how closely related a set of test items, which are supposed to be measuring a latent variable are, as a group.

**Figure 4:**
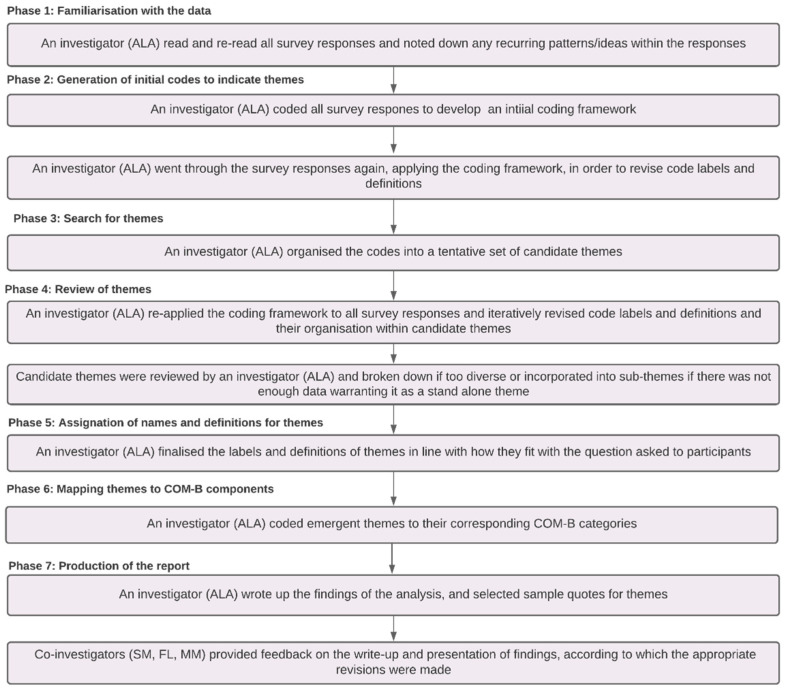
Figure showing the steps taken to analyse survey responses thematically.

Thematic analyses, in line with the approach described by Braun and Clarke [58], were used to identify: (a) reasons for not recycling food waste via council collection; (b) reason for not using compostable caddy liners and; (c) reasons why participants do not feel ready for nationwide food waste collection. Thematic analyses were conducted by the lead author—a behavioural scientist and circular economy expert—and in the steps depicted in Figure 4.

The raw survey data file exported from Qualtrics and the datasets used for the regression and thematic analyses are openly available via OSF https://osf.io/d5jw7/ (accessed on 24 February 2022).

## 3. Results

### 3.1. Participant Characteristics

In total, 1801 participants completed the survey. Participant demographics are summarised in Table 1. Participants (*M* = 56.98; *SD* = 15.49) were mostly female (65.8%) and White or White British (92.9%). The majority of participants were educated to at least undergraduate level (75.5%), privately owned their homes (86%) and lived in detached (40.1%) housing. The majority of participants were retired (42%) and preferred not to declare their annual pre-tax household income (19.3%). The size of households ranged from one to nine persons (*M* = 2.36; *SD* = 1.04) and most participants lived within a couple (42.2%) or family unit (28.9%). Our sample is representative of the UK population in terms of ethnicity [59], household annual income (It is difficult to estimate representativeness in terms of income as the majority of participants did not want to disclose this information. This interpretation is based on the results of the participants who did disclose annual household income) [60] and the number of people in the household [61], however, our sample is older [62], more educated [63] and has higher levels of home ownership [64] than national averages. Our sample is also more female and retired. Though exact figures of the returned UK population are unknown, with only 18% being over 65 [65], our sample is likely to be more retired than the wider population. Though the proportion is higher in our sample, cohabitation as a couple is also the most common type of relationship between household members in the UK [61]. Though the proportion is higher in our sample, living in a house (as opposed to a flat) is also the most common dwelling type in the UK [66].

The majority of participants (52.4%) indicated that they were being provided with a separate household food waste collection service at the time of completing the survey. This mirrors UK statistics for household food waste collection services; just under half of the households are offered such services [16]. Of these participants, the majority said that they use a separate food waste bin to recycle their food waste (85.7%). Of the participants who use a separate food waste bin to recycle food waste (*n* = 809), the majority indicated they use their food waste bin on all of the occasions that they could do so (71.2%). Additional analyses on how participants sourced their food waste bins and where they are kept in the home can be found in Supplementary File S2.

### 3.2. Factors Associated with Food Waste Recycling

#### 3.2.1. Internal Consistency of Survey

As the Cronbach’s alpha value for each COM-B domain was 0.61 or above, the items were deemed appropriate for clustering within the regression analysis.

Psychological Capability—Participants answered five items to measure psychological capability (α = 0.768), e.g., “I know what I can and can’t put in the food waste bin”, “I often forget to dispose of my food waste separately”.

Physical Opportunity—Participants answered six items to measure physical opportunity (α = 0.606), e.g., “I have sufficient space in my home for a separate food waste caddy”, “I have sufficient time to separate my food waste”.

Social Opportunity—Participants answered three items to measure social opportunity (α = 0.708), e.g., “Separating food waste is something that people I know do”, “Most people whose opinion I value would approve me of recycling my food waste”.

Automatic Motivation—Participants answered four items to measure automatic motivation (α = 0.716), e.g., “I feel guilty if I put food waste in the ordinary bin for landfill”, “Disposing of food waste separately is routine practice for me”.

Reflective Motivation—Participants answered 15 items to measure reflective motivation (α = 0.714), e.g., “I have too many things to think about other than whether or not I recycle my food waste”.

#### 3.2.2. Identification of Covariates

To avoid reducing the statistical power of our main analyses, the demographic covariates included in our regression analyses were determined by whether they had significant relationships with the outcome variable (i.e., food waste recycling behaviour).

Given that some levels of the demographic variables contained a small number of participants, some of the groups were combined or omitted in order to reduce unequal group sizes and to ensure that post hoc tests could be conducted if required. Specifically, for ethnicity, “Black or Black British”, “Asian or Asian British”, “Arab or Arab British” and “Mixed” were combined into “other”. For gender, only “Man” and “Woman” categories were used. For annual household income, “Less than £10,000 K”, “£10,000–£19,000” and “£20,000–£29,000” were combined; “£30,000–£39,000”, “£40,000–£49,000” and “£50,000–£59,000” were combined and anything higher than “£60,000–£69,000” was combined. For dwelling type “flats high rise” and “flats non-high-rise” were grouped together while “other” and “tiny home” categories were omitted. For housing type, all categories except for “owned home” were grouped as “other”. For household relationships, “sharing with friends/flatmates” was grouped with “other”. For employment status, “self-employed” and “employed” were combined while all other categories, except for “retired” were combined into “other”. For education, all degrees lower than an undergraduate degree were combined into an “up to associate degree” category. Table 2 summarises how the demographic variables were grouped and used in the subsequent analyses.

Correlational analyses and independent t-tests indicated that the number of people in the household, age, housing type and ethnicity was not associated with participants’ frequency of recycling food waste (*p*’s > 0.05). Similarly, one-way ANOVAs revealed that there were no significant differences in the frequency of recycling according to employment status or the relationship between household members (*p*’s > 0.05).

There was a significant difference in frequency of recycling according to gender *t*(929) = 2.52, *p* = 0.012, with women (*M* = 4.02; *SD* = 1.51) more likely to recycle than men (*M* = 3.7; *SD* = 1.66). There was a significant difference in frequency of recycling according to education F(2, 941) = 4.821, *p* = 0.008, *η_p_^2^* = 0.01. Pairwise comparisons (with Bonferroni adjustment) revealed participants with postgraduate degrees (*M* = 3.75; *SD* = 1.63) were less likely to recycle food waste than those with undergraduate degrees (*M* = 4.11; *SD* = 1.45). There was also a significant difference in recycling frequency according to income F(2, 739) = 4.62, *p* = 0.01, *η_p_^2^* = 0.01, and dwelling type F(3, 933) = 3.30, *p* = 0.2, *η_p_^2^* = 0.01. Pairwise comparisons (with Bonferroni adjustment) revealed that participants with incomes in the £30,000–£59,000 range (*M* = 4.14; *SD* = 1.41) were more likely to recycle than those in the £10,000–£29,000 range (*M* = 3.74; *SD* = 1.64) and participants living in terraced housing (*M* = 3.74; *SD* = 1.61) were less likely to recycle than participants in semi-detached housing (*M* = 4.15; *SD* = 1.43). Hence four demographic variables were controlled for in subsequent analyses: gender, education, income, and dwelling type.

#### 3.2.3. Predicting Food Waste Recycling Behaviour

The relevant assumptions of this statistical analysis were met. Specifically, an analysis of standard residuals was carried out, which showed that the data contained no outliers (Std. Residual Min = −2.43, Std. Residual Max = 2.63). Collinearity statistics (i.e., Tolerance and VIF) were all within accepted limits [67] (Psychological Capability, Tolerance = 0.37, VIF = 2.70; Social Opportunity, Tolerance = 0.67, VIF = 1.50; Physical Opportunity, Tolerance = 0.60, VIF = 1.66, Automatic Motivation, Tolerance = 0.40, VIF = 2.52, Reflective Motivation, Tolerance = 0.43, VIF = 2.31). If the VIF values are close to 1, this indicates that the predictors are not correlated; when 1 < VIF < 5 the predictors are moderately correlated; if the values are greater than 5–10 (the predictors are highly correlated), this suggest that the regression coefficients are poorly estimated [68]. The data met the assumption of independent errors (Durbin–Watson value = 1.98). Residual and scatter plots indicated the assumptions of normality, linearity and homoscedasticity were all satisfied. The data also met the assumption of non-zero variances.

Using the enter method, a two-stage hierarchical regression was conducted with food waste recycling behaviour as the dependent variable. Gender, education, income and dwelling type were entered in the first stage to control for these factors. COM-B factors were entered at the second stage. Descriptive statistics for the study variables are reported in Table 3. Intercorrelations between the continuous multiple regression variables are reported in Table 4 and the regression statistics are in Table 5.

The hierarchical multiple regression revealed that at stage one gender, income, education and house structure accounted for 10.5% of the variation in food waste recycling behaviour but did not significantly contribute to the regression model F(4, 737) = 2.025, *p* = 0.089. Adding COM-B components in stage two significantly contributed to the regression model by explaining 39% of the variation in participants’ food waste recycling F(9, 737) = 14.54, *p* < 0.001. Inspection of the beta weights revealed that participants’ automatic motivation and psychological capability were associated with a significant increase in the frequency of food waste recycling while education was significantly associated with a decrease in the frequency of food waste recycling.

### 3.3. Reasons for Not Recycling Food Waste via Council Food Waste Collection

Those who said “no” to recycling their food waste via local services provided reasons for their answer. Eight themes emerged from the responses, relating to physical opportunity (*n* = 6), psychological capability (*n* = 1) and automatic motivation (*n* = 1). These are summarised in Table 6 with frequencies and example quotes.

The most popular method for dealing with food waste mentioned was home composting followed by feeding any leftover food waste to their pets (e.g., dogs, chickens) or leaving it out for nearby wildlife and strays. Others said that they put any food waste in with garden waste to be collected. Two participants said that they use their neighbours’ bins when they need to. Another reason mentioned by participants for not using food waste collection was not producing any or producing minimal food waste in the first place. Relating to this, following a predominantly plant-based diet (both vegan and vegetarian) was attributed to not needing to use council waste collection services as participants were able to manage plant-based food waste at home.

Hygiene (e.g., bacteria), odour (e.g., bad smell) and pest concerns relating to keeping food waste in the house were mentioned. The cost was also a barrier to recycling food waste via council waste collection both in terms of financial and time costs. For example, participants mentioned that it was too much effort and hassle to recycle food waste, particularly for those who produce little food waste anyway; for these participants recycling food waste was not seen as justifiable. Related to cost, participants mentioned the high cost of buying separate compostable caddy liners for a food waste bin. Participants also mentioned service-related barriers to using council food waste collection. These include unreliable (e.g., late) and inadequate (e.g., the council only provides non-compostable bags) food waste collection services.

Lack of space for bins was a barrier to recycling food waste. For example, this included no space within the home due to living in a small flat or having a small kitchen. Participants also mentioned that there is not enough space outside their home to fit an extra bin for food waste on top of the existing bins for recycling and landfill. Other participants mentioned not having control over household management (e.g., living at home with parents who manage household waste and decide to not recycle food) and not having a separate food waste bin at home in the first place.

Lack of knowledge and awareness was also a barrier identified. For example, some participants did not understand why recycling food waste is necessary or environmentally beneficial while others indicated that they did not know how to go about recycling their food waste.

### 3.4. Reasons for Not Using Compostable Caddy Liners

Those who said “no” to using compostable caddy liners provided reasons for their answer. Ten themes emerged from the responses, relating to physical opportunity (*n* = 6) and reflective motivation (*n* = 3) and psychological capability (*n* = 1). These are summarised in Table 7 with frequencies and example quotes.

Repurposing other types of bags/materials was the main reason offered by those not using compostable bags. There was variation in the responses in terms of the different types of materials used in place of compostable caddy liners. Participants reported using other types of materials including newspaper or repurposing other types of packaging such as paper bags, paper towels, magazine wrappers and plastic shopping bags for the purpose of lining their food waste bins.

Factors relating to local councils’ food waste collection capabilities were also frequently mentioned such as: (a) their council accepts non-compostable liners for food waste collection; (b) the council does not provide compostable bags freely; (c) their council has explicitly stated that they do not want compostable caddy liners to be used; (d) council provides non-compostable caddy liners, and (e) that waste collectors do not collect their food waste if it is placed out for collection in a compostable bag as they think that it is plastic.

Accessibility was also an issue. For most participants, this manifested as barriers relating to cost—having to buy compostable caddy liners were reported as an additional expensive household cost. Another participant mentioned that disability prevents them from being able to access shops that stock compostable caddy liners. Availability was also an issue in that participants reported not being able to find compostable caddy liners in stores locally.

Competing priorities such as the perceived inconvenience of using an extra caddy liner was another issue. Relating to this, participants also reported feeling that there is no need for an additional caddy liner or that it was just as easy to clean the bin directly and so choose not to use them. Beliefs about the environmental impacts of caddy liners was also an issue perceiving compostable caddy liners as wasteful in their own right.

Design related issues such as size and durability were mentioned. Compostable caddy liners were reported to be too small and fragile. They reportedly tear too easily, producing more waste and making them more expensive. Not knowing that compostable caddy liners existed but also not knowing where to find them was also an issue.

### 3.5. Reasons for Not Feeling Ready for Nationwide Food Waste Collection

Although the majority (73.9%) of participants had not heard of the UK government’s plan to implement separate food waste collection services nationwide by 2023, the majority (84.6%) of participants reported feeling ready for these changes (Table 1). Those who did not feel ready or were unsure provided a reason for their answers. Seven themes emerged relating to psychological capability (*n* = 2), physical opportunity (*n* = 1), automatic motivation (*n* = 1) and reflective motivation (*n* = 3). These are summarised in Table 8 with frequencies and example quotes.

Many participants expressed that their lack of readiness was due to not knowing what such a scheme would mean for them and their households. For example, this included not knowing whether there would be penalties for not using the service (e.g., if they prefer to home-compost their waste), what types of food waste the scheme would accept and whether they would have to pay additional costs for this service (e.g., via the raising of their council tax, additional caddy liner costs).

Participants reported lacking the space to take on the additional responsibility of recycling their food waste including mental “headspace” (i.e., the additional burden of having to take on a new recycling responsibility) and physical space in their homes or kerb-side for an additional bin. Participants also mentioned no need for the scheme as a reason behind why they did not feel ready for it either because they produce little food waste or prefer to deal with their waste in other ways (e.g., via home-composting).

Participants expressed concerns with respect to the hygienic storage of food waste between collections. This concern manifested both within the home and outside within the local community. Within the home, there were concerns with respect to the smell of rotting food waste and the waste attracting pests, such as rats and flies. There were also similar concerns of bad smells around the outside bins and food waste bins attracting cats, dogs, foxes and other animals that look through the bins and scatter their contents on the streets and polluting the neighbourhood. Participants also mentioned that an extra food waste bin outside, in addition to all the other bins residents must have outside their homes, would cause clutter.

Participants, particularly those living in multi-occupancy buildings (e.g., estates and flats), expressed implementation concern about the logistics and practicalities of rolling out such a scheme citing a lack of faith in their local council to be able to offer such a service efficiently.

Pessimism over the success of such a scheme was reported as a reason for not feeling ready for food waste collection services as was a lacked knowledge of what food waste items can be recycled.

## 4. Discussion

This study aimed to identify influences on food waste recycling amongst UK citizens; the reasons why citizens with access to food waste collection services do not recycle food waste; the reasons why citizens who do recycle do not use compostable caddy liners, and; the reasons why citizens feel unprepared for UK-wide food waste collection services.

Quantitative findings showed that psychological capability and automatic motivation were significant predictors of recycling behaviour. Having a higher annual household income, identifying as female, being less educated and living in semi-detached (vs. terraced) housing and made citizens more likely to recycle food waste. Physical opportunity (i.e., dealing with food waste in other ways such as home-composting or feeding pets/strays, time and financial costs) was the main barrier to recycling food waste identified in qualitative analyses. Participants also reported automatic motivation related barriers such as concerns over pests, odour and/or hygiene and local authorities’ food waste collection capabilities.

Barriers to using compostable caddy liners included: engaging in conflicting behaviours such as cleaning the bin out after each use or repurposing other materials such as newspaper (physical opportunity), council related barriers such as the council not accepting compostable liners (physical opportunity), low availability and accessibility of compostable liners (physical opportunity) and the perception that compostable liners are unnecessary and wasteful themselves (reflective motivation). 

Participants reported feeling unprepared for nationwide food collection services due to lack of scheme awareness (psychological capability), not having the time or space for taking on the extra responsibility (physical opportunity) and a lack of confidence that there is a public need for food waste collection services (reflective motivation). Concerns relating to pests, pollution and implementation (automatic motivation) were also reported.

These results support and extend prior findings. There is ample research showing that higher-income households [69,70] and women are more likely to recycle [71]; our study suggests that this also extends to food waste. The higher rates of recycling by citizens living in semi-detached (vs. terraced housing) could owe to these homes, generally, being more spacious, e.g., by allowing for more bin space both inside and outside the home—our qualitative findings support the lack of household space as a barrier to recycling.

Some results contradict prior findings. Higher levels of education are, in general, found to positively influence recycling behaviours [70,72,73] whereas we found higher levels of education to decrease recycling. Given the older age, majority retired and higher-income nature of the sample, our results could be explained by the fact that those who were more educated were more likely to engage in home-composting and so less likely to recycle food waste using local services; older age, being retired, higher household income and higher education are all factors associated with engagement in home-composting [74,75]. As we did not collect data on participant home-composting status, it is difficult to ascertain whether this factor influenced our results. We recommend that future studies collect this data and control for the home-composting status of participants in subsequent analyses.

The significant association of automatic motivation and psychological capability with behaviour are expected findings. Automatic motivation related behavioural influences including emotions, such as guilt, and habits have previously been identified as predictors of both recycling behaviours [76,77] and household food waste management behaviours [78,79]. Disgust was identified, across a variety of countries and contexts, not only as a key emotion specific to deterring handling of food waste [80,81] but also as an emotion important to understanding food waste behaviours, more generally [78,82,83]. Psychological capability related barriers such as lack of knowledge (i.e., knowing which bin to what which waste items in) were associated as barriers to related organic waste recycling behaviours such as disposal of compostable plastic packaging [38,84] and recycling of waste materials more generally [85]. The salience of physical opportunity as a key barrier across the qualities findings is also in line with prior findings. Other studies report the lack of necessary infrastructure to participate in household waste recycling as one of the most important barriers for households not to participate in recycling activities [86]. In addition, participants’ reasons for feeling unprepared for nationwide food collection (i.e., concerns relating to space, hygiene, implementation and management) are echoed in other similar studies investigating barriers to engagement with food waste recycling schemes [87].

### Implications, Limitations and Future Research

There are strong practical and theoretical implications to this study. Our findings reiterate that a combination of capability, opportunity and motivation are required to enact behaviour. Theoretically, this is consistent with the COM-B model’s assertion of behavioural determinants which is important if they are to form the basis of intervention development. Practically, the implications for intervention design are clear—holistic approaches are needed to promote the adoption of necessary behaviours.

Getting citizens engaged in food waste recycling schemes is a major concern for local UK authorities [88]. It is hoped that our results can help inform behaviour change interventions that will lead to a successful nationwide food waste collection and recycling strategy. In terms of intervention design, our results suggest that strategies increasing citizen’s automatic motivation (e.g., making recycling food waste a regular household routine and reducing the perceived “unpleasantness” associated with handling food waste) and psychological capability (e.g., increasing knowledge of what items can go into their food waste bins) are likely to be effective at getting citizens to separate their food waste. This could involve the development of clear, consistent communications aimed at increasing knowledge of what can and cannot be put in food waste bins (which, in turn, would benefit from consistency in the items that can be collected and processed across regions). It could also involve the improved functional design and free distribution of bins (e.g., well-ventilated) and compostable caddy liners (e.g., that do not disintegrate), developed according to user-centred needs for cleanliness, convenience and hygiene. Financial incentives can also be an effective intervention strategy for getting people to start recycling, particularly for “low emotional involvement” items (which household food waste is assumed to be) [88]. These types of incentives, e.g., cash, coupons or lotteries, may be an effective way of turning a behaviour that was not routine in the first instance, into one that is, which can then be sustained, in the long term, by habit. Rewards and repetition are key habit-forming mechanisms [89] that financial incentives can enable, though findings are mixed regarding sustained behaviour change when incentives are removed [90,91].

Our results reveal a lack of faith in local authorities’ ability to collect the waste regularly and efficiently. There also appears to be inconsistent messaging from local authorities with respect to compostable caddy liner use—some residents are asked to use them while others are told not to. At present, there also appears to be concern from UK citizens about whether implementing nationwide food waste collection and recycling services is a meaningful and useful use of public funds. To increase the likelihood of successful implementation, our results suggest that a UK-wide food waste collection service may benefit from ensuring it is nationally uniform (to increase consistency), efficient (to increase engagement) and reliable (to increase trust). It is therefore imperative that alongside increasing local councils’ physical capabilities to collect food waste, there are public media campaigns aimed at not only increasing the public’s confidence in local council’s capabilities but also highlighting the importance of food waste recycling for public and environmental health to shift the perception of the value of this service.

Outside of the present, specific UK food waste context, there is also a great potential utility for this study to provide insight and guidance to other researchers and practitioners. The benefits of using integrative theoretical frameworks (e.g., COM-B) in citizen behaviour change research include an improved understanding of the factors influencing behaviour. When this evidence is applied to intervention development (e.g., by applying the wider BCW intervention development framework of which COM-B is a part of [25,26]), this leads to the design of behaviour change strategies that are more likely to be effective. Our study provides a guide and adaptable template that can be used by others to design such theoretically-informed interventions.

A limitation of this study is that we did not collect data on whether participants engaged in home-composting. Given that one of the sampling techniques was via a home-composting citizen science experiment mailing list, it is likely that home-composting status would have impacted whether or not one recycled via local collection services; this was also the top reason provided for not recycling food waste via local collection services. Collecting these data would have allowed us to control for this factor in our regression analyses. In addition, given the self-selecting and voluntary nature of participation, this study may have attracted more “pro-environmental” participants and those more likely to recycle food waste. This is exemplified by the fact that although many participants had not heard of the UK’s plan to implement nationwide food waste collection, the majority had indicated feeling ready for it. Recycling behaviours are morally relevant [92] and often exaggerated behaviours [93]. It is therefore plausible for this study to have suffered social desirability bias as exemplified by the fact that the majority of participants indicated that they “always” put food waste in the food waste bin. Though a recent meta-analysis has shown the relationship between social desirability and pro-environmental intentions and behaviours to be small [94], our results should be interpreted with these potential limitations in mind.

While our results provide insight into behavioural influences, further studies are required to replicate the findings and assess their generalisability to other segments of the UK population. While ensuring that samples are representative in terms of socio-economic demographics, we recommend also ensuring they are representative in terms of “psychological” demographics, e.g., environmental orientation, political leaning, etc. We believe this is particularly important for environmental research where such variables may influence results. There are evidence-based toolkits designed to support this process. For instance, market segmentation of the British public was published by Climate Outreach in its Britain Talks Climate report [95]. Based on research and stakeholder consultation, this report segments the British public into seven possible “psychological” groups using a range of ideological and psychological factors which provides insight on how they might engage with issues relating to climate change. Where ensuring representativeness in terms of psychological demographics is not feasible, it is important that studies collect this data that will allow for psychological segmentation. It will result in better sample description, the ability to control for these variables in analyses and better contextualisation of results. In turn, knowing whether the barriers and enablers identified in our study are corroborated within other populations, e.g., those with less pro-environmental intentions, will help to clarify the robustness of our findings. We also recommend further research to investigate the behaviour of other key actors within the food waste recycling system, e.g., local councils. This will allow for a more holistic, systems understanding of the barriers and enablers to recycling food waste which, in turn, will allow for the design of interventions more likely to be effective.

## 5. Conclusions

Implementation of a nationwide food waste collection strategy relies not only on developing the appropriate infrastructure but also on citizens adopting the necessary set of food waste recycling behaviours. In terms of developing a UK-relevant behaviour change strategy to promote adoption of food waste recycling, our study emphasises the importance of increasing citizens’ automatic motivation and psychological capabilities to recycle food waste, for instance, by increasing knowledge of what can be recycled, awareness of the importance of recycling food waste and designing products that reduce the perceived unpleasantness of dealing with food waste (e.g., functionally designed bags and bins). It also emphasises the need to reduce existing physical opportunity related barriers, for example, by providing appropriate bins and bin liners free of charge and increasing the reliability and efficiency of waste collection services. Further research on this topic is needed to assess the consistency of results amongst other UK demographics, e.g., younger populations. Additional studies controlling for home-composting status will help promote the conclusiveness of results. Although UK-focussed, our results have valuable implications for policy and intervention design. We provide a methodological framework for conducting behavioural analyses that can aid in designing interventions more likely to be effective at achieving sustainable behaviour change. The practical usefulness of our study includes the detailing of a method that can be adapted by other researchers and practitioners investigating similar waste management behaviours.

## Figures and Tables

**Table 1:** Table summarising participant demographics.

Characteristics	N (Missing)	%	Mean (*SD*)
Gender	1801 (0)		
Male	593	32.9	
Female	1185	65.8	
Non-binary	5	0.3	
Prefer not to say	18	1	
Age (years)	1763 (38)		56.98 (15.49)
Ethnicity	1790 (11)		
White or White British	1674	92.9	
Arab or Arab British	2	0.1	
Asian or Asian British	40	2.1	
Black or Black British	21	1.2	
Mixed	32	1.8	
Any other ethnic background	21	1.2	
Highest level of education	1801 (0)		
Primary education	1	0.1	
Lower secondary education	56	3.1	
Higher secondary education	164	9.1	
Vocational certificate	157	8.7	
Associate degree	59	3.3	
Undergraduate degree	678	37.6	
Postgraduate degree	474	26.3	
PhD/ Doctorate	212	11.8	
Employment status	1801 (0)		
Retired	773	42	
Employed	653	36	
Self-employed	191	10.6	
Homemaker	49	2.7	
Student	44	2.4	
Out of work (looking for work)	24	1.3	
Unable to work	23	1.3	
Out of work (not looking)	15	0.8	
Other	29	1.6	
Recruitment Method	1801 (0)		
Social media/email	1501	83.3	
Prolific	300	16.6	
Annual household income pre-tax	1801 (0)		
Less than £10,000	61	3.3	
£10,000 to £19,999	207	11.5	
£20,000 to £29,999	255	14.2	
£30,000 to £39,999	224	12.4	
£40,000 to £49,999	163	9.1	
£50,000 to £59,999	137	7.6	
£60,000 to £69,999	98	5.4	
£70,000 to £79,999	80	4.4	
£80,000 to £89,999	58	3.2	
£90,000 to £99,999	32	1.8	
£100,000 to £149,999	97	5.2	
£150,000 or more	41	2.3	
Prefer not to say	348	19.3	
Housing type	1801 (0)		
Owned home	1549	86	
Privately rented	177	9.8	
Council housing	40	2.2	
Student accommodation	6	0.3	
Other	29	1.6	
Dwelling type	1801 (0)		
Detached	738	40.1	
Semi-detached	551	30.6	
Terraced	318	17.6	
Flats non-high rise	152	8.4	
Flats high rise	13	0.7	
Tiny home	7	0.3	
Other (e.g., boat home)	22	1.2	
Number of people in household	1766 (35)		2.36 (1.04)
Household relationships	1801 (0)		
Couple	760	42.2	
Family	701	28.9	
Single person	265	14.7	
Sharing with friends/flatmates	46	2.6	
Other	29	1.6	
Food waste collection services available	1801 (0)		
Yes	944	52.4	
No	830	46.1	
Unsure	27	1.5	
If YES, use of a food waste caddy	944 (0)		
Yes	809	85.7	
No	135	14.3	
If YES, frequency of caddy use and;	809 (0)		
Always	577	71.2	
Most of the time	123	15.2	
About half the time	23	2.8	
Sometimes	74	9.1	
Never	12	1.5	
If YES, use of compostable caddy liners	809 (0)		
Yes	555	68.6	
No	185	22.8	
Sometimes	69	8.5	
Awareness 2023 food waste scheme	1801 (0)		
Yes	375	20.8	
No	1331	73.9	
Not sure	95	5.3	
Readiness for 2023 food waste scheme	1801 (0)		
Yes	1523	84.6	
No	134	7.4	
Not sure	144	7.9	

**Table 2:** Table summarising demographic variables used in the regression analyses.

Variable	N (Missing)	Percentage %
Ethnicity	937 (7)	
White or White British	875	93.3
Other	62	6.6
Gender	931 (13)	
Woman	649	69.7
Man	282	30.3
Annual household income pre-tax	742 (202)	
£10,000–£29,000	258	34.8
£30,000–£59,000	260	35
£69,000 +	224	30.2
Housing type	944 (0)	
Owned home	836	88.6
Other	108	11.4
Dwelling type	937 (7)	
Detached	386	41.2
Semi-detached	285	30.4
Terraced	193	20.6
Flat	73	7.8
Household relationships	944 (0)	
Couple	381	40.4
Family	384	40.7
Single	38	4
Other (e.g., flat-share)	141	14.9
Employment	944 (0)	
Retired	408	43.3
Employed/self-employed	443	46.9
Other (e.g., student)	93	9.9
Education	944 (0)	
Up to associate degree	226	23.9
Undergraduate degree	362	38.3
Postgraduate degree	356	37.7

**Table 3:** Descriptive statistics for study variables.

Variables	N	Mean	*SD*	Min	Max
Recycling behaviour	738	3.91	1.57	1.00	5.00
Gender	738	1.32	0.48	1.00	2.00
Income	738	1.96	0.8	1.00	3.00
Education	738	2.17	0.76	1.00	3.00
House structure	738	1.98	0.98	1.00	4.00
Psychological capability	738	4.75	0.54	1.00	5.00
Social opportunity	738	4.18	0.74	1.00	5.00
Physical opportunity	738	4.48	0.57	1.00	5.00
Automatic motivation	738	4.43	0.81	1.00	5.00
Reflective motivation	738	4.12	0.39	1.00	5.00

**Table 4:** Correlations between study variables. All were significant at *p* < 0.001 **.

Variables	1.	2.	3.	4.	5.	6.
1. Recycling behaviour	-	0.339 **	0.189 **	0.204 **	0.351 **	0.256 **
2. Psychological capability	-	-	0.384 **	0.618 **	0.722 **	0.599 **
3. Social opportunity	-	-	-	0.308 **	0.373 **	0.566 **
4. Physical opportunity	-	-	-	-	0.486 **	0.431 **
5. Automatic motivation	-	-	-	-	-	0.655 **
6. Reflective motivation	-	-	-	-	-	-

**Table 5:** Regression of COM-B factors on frequency of food waste recycling behaviour.

Covariates/Predictors	β	*t*	sr^2^	*R*	*R* ^2^	∆*R*^2^
Step 1				0.105	0.001	0.006
Gender	−0.037	−10.062	0.071			
Income	0.067	10.912	0.071			
Education	−0.08 *	−20.294	−0.085			
Structure of housing	0.048	10.363	0.05			
Step 2				0.390	0.152	0.142
Psychological Capability	0.188 ***	30.345	0.123			
Social Opportunity	0.058	10.378	0.051			
Physical Opportunity	−0.019	−0.433	−0.016			
Automatic Motivation	0.233 ***	40.298	0.157			
Reflective Motivation	−0.026	−0.510	−0.019			

Notes. *N* = 738; β = standardised beta coefficients; *t* = *t*-test value; sr^2^ = semi-partial correlation coefficient; *p* = significance; ∆*R*^2^ = adjusted *R*^2^. * *p* < 0.05, *** *p* < 0.001.

**Table 6:** Frequency of themes alongside example quotes depicting reasons for not recycling food waste via local services.

COM-B	Themes	Sub-Themes	Example Quotes
Physical opportunity	Recycles food waste in other ways (*n* = 97)	- does home composting (*n* = 76) - feeds food waste to pets/local wildlife (*n* = 10) - puts it in garden waste (*n* = 9) - uses neighbour’s bin (*n* = 2)	*“I compost it myself”;* *“…anything we do not eat goes to birds, foxes/strays...”* *“It goes in the green garden waste bin, together with perennial weeds, woody garden waste, etc.”;* *“Very very occasionally we put something (such as meat bones, which we seldom have) into our neighbours’ food recycling bin.”*
Physical opportunity	Produces no/minimal food waste (*n* = 20)		*“I don’t produce any food waste”*
Automatic motivation	Pests/hygiene (*n* = 18)	- Smell/hygiene (*n* = 9) - Pests (*n* = 9)	*“The smell and hygiene associated.”;* *“Our previous experience is that it attracts a lot of flies and unsanitary bacteria to the house.”;* *“I [don’t] want it to make the house smell or attract mice/rats etc”*
Physical opportunity	Follows plant-based diet (*n* = 17)		*“Because we eat a vegetarian diet, we put all our food waste in the bin”*
Physical opportunity	Cost (*n* = 15)	- too much effort/hassle to recycle food waste (*n* = 12) - compostable bags perceived as too expensive (*n* = 3)	*“It’s too much hassle”; “[I] live alone and don’t produce enough food waste to make it worthwhile”;* *“Living in a small top floor flat, it’s not convenient to store the food waste bin in a small kitchen and have to carry it down stairs. No one in the building (four flats) uses any of the food waste bins—I think for similar reasons.”* *“The bags the council insist we use inside our food waste bins are so expensive!”*
Physical opportunity	Service-related factors (*n* = 12)	- unreliable food waste collection services (*n* = 9) - council does not provide free food waste bins (*n* = 2) - council only provides non- compostable bags (*n* = 1)	*“It’s never collected and it stinks”;* *“We are not given a bin for food waste where we live.”;* *“Our council supply us with single use polythene bags as liners, rather than compostable material”*
Physical opportunity	Household related factors (*n* = 10)	- no space within home (*n* = 6) - no space for an extra food waste bin outside (*n* = 2) - no bin at home (*n* = 1) - not responsible for household decisions (*n* = 1)	*“We don’t have space in our (rented) kitchen for an additional bin on top of the general and recycling bins.”;* *“I have room for 3 wheelie bins outside my house, but I have 4 bins. I have chosen to leave the food/garden waste bin out of the way in my garage.* *It therefore doesn’t get used……”;* *“We don’t have a separate food waste bin”* *“My parents manage the food waste and they have chosen not to do so. I do not know their reasons for this.”*
Psychological capability	Lack of knowledge/awareness (*n =* 4)	- does not understand point of recycling (*n =* 3) - lacks knowledge on how to recycle food waste (*n =* 1)	*“We just never have, no reason really, would be good to know what happens to food waste what are the benefits of putting it in a separate bin?”;* “*not sure how to.”*

**Table 7:** Frequency of themes alongside example quotes depicting reasons for not using compostable liners.

COM-B	Themes	Sub-Themes	Example Quotes
Physical opportunity	Repurposing other types of bags/materials (*n* = 75)		*“I use paper bags”* *“We wrap our food waste in newspaper.”* *“Stainless steel container and use paper towelling to line it.”*
Physical opportunity	Council-related factors (*n* = 50)	- council accepts non-compostable liners (*n* = 38) - council does not provide them freely (*n* = 5) - council does not want them to be used (*n* = 6) - council provides non-compostable caddy liners (*n* = 14) - collectors leave waste thinking it is wrapped in plastic (*n* = 1)	*“our local council recycling scheme says we can use any bag for recycling food waste”* *“They are not provided by the local council, we are a very low income family so its an extra expense we don’t need.”* *“Our local council do not accept any type of compostable plastic in with the food waste.”* *“Council provides plastic bags for the purpose—they switched to plastic from starch 2 years ago”* *“Waste collectors think it is regular plastic and will not collect the bin until the bag is removed”*
Reflective motivation	Lack of necessity (*n* = 49)		*“Unnecessary additional waste”* *“No need for a bin liner”* *“I don’t believe it’s necessary to spend anything further to enable me to dispose of waste.”*
Physical opportunity	Accessibility (*n* = 27)		*“…because I am disabled, I cannot always get to the correct supermarket that sells the right type & size of bin liner”* *“The price”* *“Quite expensive”*
Reflective motivation	Beliefs about environmental impacts (*n* = 21)		*“Even though they are compostable they still are bad for the environment. They require carbon to manufacture, transport etc and I’m not entirely sure whether they break down in “* *“…unsure if materials break down as easily as they should”* *“I use paper bags instead. That way the paper bags are used twice. (and I do not need to use specially made bin liners at all) I believe this is less wasteful.”*
Physical opportunity	Cleans food waste bin directly (*n* = 21)		*“We simply wash the container”* *“In my kitchen bin I use no liner at all, just regularly brush the material into the council bin ready to go out to the kerb later.”* *“I did not find the compostable food caddy liners you can buy particularly helpful—it is just as easy to empty the food bin into the one council collect.”*
Physical opportunity	Availability (*n* = 15)		*“Not always available”* *“My local shop doesn’t sell them”*
Physical opportunity	Design-related factors (*n* = 12)		*“The bin liners are very fragile and tear a lot, wasting bags. I have to pay for them (to not be suitable for what I need)”* *“They are not always available or big enough”*
Psychological capability	Lack of knowledge/awareness (*n* = 6)		*“I don’t know where to get them from”* *“Wasn’t aware of them or how to use them”*
Reflective motivation	Priorities (*n* = 6)		*“Inertia. I’ve not got around to sourcing any.”* *“I use the depending on what I’m putting in it to make the bin easier to fully empty into my garden waste/food recycling bin.”* *“Inconvenience and cost of maintaining supply of compostable bags.*

**Table 8:** Frequency of themes alongside example quotes depicting readiness for nationwide food waste collection.

COM-B	Themes	Sub-Themes	Example Quotes
Psychological capability	Scheme awareness/clarity (*n* = 93)		*“Ready in principle, but until details are made public it’s hard to know how it will work for our household”* *“We have not yet been provided with any information about how this will work in my local area.”*
Physical opportunity	Space for the additional responsibility (*n* = 36)	- headspace (*n* = 8)	*“Sounds like a lot of hassle”* *“I think my household are currently unprepared as this is something we do not do at the moment and would need to get into the routine of doing.”* *“…If it means more bins cluttering streets and gardens I should not be pleased”* *“Limited space in my kitchen already used for normal waste, paper, home compost, glass. So how do [I] organise space for yet another bin?”*
Reflective motivation	Public need for the scheme (*n* = 35)		*“As an older person I have next to nothing to send to a food waste collection so for me it would be a waste of resources.”* *“We produce such a small amount of food waste it would generally mean putting out a container with next to nothing in it.”* *“I currently compost what little food waste that we have and I would very much object to being mandated to change that very satisfactory method.”*
Automatic motivation	Pests and pollution (*n* = 33)		*“I see reservations re pollution and smells”* *“I think [it’s] a great idea, but it is difficult to do. I live in an area with a lot of foxes and other animals that look through the bin and scatter the contents which makes me somewhat hesitant.”* *“I would like more information. I would wish to [know] the containers were well sealed and very regular and definite collections before I agree”*
Reflective motivation	Implementation concerns (*n* = 20)	- lack of trust in council (*n* = 8)	*“Multi occupancy buildings have challenges with dealing with this and the necessary infrastructure may not be available to support its implementation.”* *“I personally am ready and willing to recycle food waste separately, however the block of flats that I currently live in do not currently provide recycling bins for other recycling such as plastic or card.* *Due to this I feel that I would not be ready or able to recycle food waste.”* *“Our local council does not supply any recycling facilities at all for our block of flats, and has not done so for over a year.”*
Reflective motivation	Pessimism (*n* = 5)		*“I am not sure if this would actually make a difference”* *“People still won’t bother”*
Psychological capability	Knowledge (*n* = 3)		*“Not sure what can go in food waste recycling.”* *“…Bit more training on what goes in would be nice.”*

## Data Availability

Data used in this study can be found on Open Science Framework: https://osf.io/d5jw7/ (accessed on 24 February 2022).

## Supplementary Materials

The Thematic Codebook and Data Saturation Table are available online at https://www.mdpi.com/article/10.3390/ijerph19052729/s1, File S1: Table mapping survey items to theoretical constructs [96,97,98,99,100,101,102,103,104,105,106], File S2: Supplementary analyses.

## Abbreviations

AD	Anaerobic digestion
IVC	In Vessel Composting
COM-B	Capability–Opportunity–Motivation–Behaviour
BCW	Behaviour Change Wheel
TDF	Theoretical Domains Framework
OSF	Open Science Framework

## Appendix A

**Table A1:** Name and Definition of TDF Domains [28].

TDF Domain	Explanation
Knowledge	An awareness of the existence of something
Skills	An ability or proficiency acquired through practice
Social/Professional role and identity	A coherent set of behaviours and displayed personal qualities of an individual in a social or work setting
Beliefs about capabilities	Acceptance of the truth, reality or validity about an ability, talent or facility that a person can put to constructive use
Optimism	The confidence that things will happen for the best or that desired goals will be attained
Beliefs about consequences	Acceptance of the truth, reality, or validity about outcomes of behaviour in a given situation
Reinforcement	Increasing the probability of a response by arranging a dependent relationship, or contingency, between the response and a given stimulus
Intentions	A conscious decision to perform a behaviour or a resolve to act in a certain way
Goals	Mental representations of outcomes or end states that an individual wants to achieve
Memory, attention and decision processes	The ability to retain information, focus selectively on aspects of the environment and choose between two or more alternatives
Environmental context and resources	Any circumstance of a person’s situation or environment that discourages or encourages the development of skills and abilities, independence, social competence and adaptive behaviour
Social influences	Those interpersonal processes that can cause individuals to change their thoughts, feelings, or behaviours
Emotion	A complex reaction pattern, involving experiential, behavioural, and physiological elements, by which the individual attempts to deal with a personally significant matter or event
Behavioural Regulation	Anything aimed at managing or changing objectively observed or measured actions
