# Supplementary material for: Barriers and Enablers to Food Waste Recycling: A Mixed Methods Study amongst UK Citizens
Source: Int J Environ Res Public Health. 2022 Feb 26;19(5):2729. doi: 10.3390/ijerph19052729 (PMC8910430; doi:10.3390/ijerph19052729)
Supplement: Supplementary file 1 [file ijerph-19-02729-s001.zip › S1. Survey items.pdf]

Supplementary File S1. Table showing questionnaire items, related constructs and COM-B domains

COM-B domain	Construct	Survey Item	Rationale
Psychological Capability (α = 0.768)	Memory (2)	1. I often forget to dispose of my food waste separately<br>2. I have trouble remembering to put food waste into a separate bin	Adapted from O'Brian et al.[1], Cane et al.[2] and Michie et al.[3]
	Knowledge (2)	3. I know what I can and can't put in the food waste bin<br>4. I know whether or not separate food waste collection is available to me	Adapted from Taufik et al. [4], Cane et al.[2] and Michie et al.[3]
	Self-efficacy (1)	5. Disposing of food waste separately is easy for me	Adapted from Bandura et al.[5]
Physical Opportunity (α = 0.606)	Resources/material (6)	6. I have sufficient space in my home for a separate food waste caddy<br>7. I find food waste caddies to be too small<br>8. I have sufficient time to separate my food waste<br>9. Buying a separate food waste caddy for my home is too expensive<br>10. I don't have an appropriate container that I could use as a food waste caddy<br>11. Food waste caddies are not available to me free of charge	Adapted from Allison et al.[6], Oliveira et al. [7] Cane et al.[2] and Michie et al.[3]
Social Opportunity (α = 0.708)	Subjective norms (2)	12. Most people who are important to me think that I should separate my food waste<br>13. Most people whose opinion I value would approve me of recycling my food waste	Adapted from Ajzen et al.[8] and Khan et al.[9]
	Descriptive norms (1)	14. Separating food waste is something that people I know do	Adapted from Wakefield et al.[10] and Cialdini et al.[11]
Automatic Motivation (α = 0.716)	Automaticity (3)	15. I'm in the habit of disposing my food waste into a separate food waste bin<br>16. Disposing my food waste separately is something that I do automatically<br>17. Disposing of food waste separately is routine practice for me	Adapted from Russell et al.[12] and Verplanken et al.[13]

	Memory, Attention, Decision Making (1)	18. Separating my food waste isn't really something I think about	Cane et al.[2] and Michie et al. [3]
	Affect (2)	19. I feel guilty if I put food waste in the ordinary bin for landfill	Adapted from Wakefield et al. [10] and Russell et al.[12]
Reflective Motivation (α = 0.714)	Action planning (1)	20. I have a clear plan of how I will dispose of my food waste separately	Adapted from Sniehotta et al.[14]
	Outcome expectancies (2)	21. If I recycle food waste, it will be good for the environment<br>22. I think my recycling food waste is too small a gesture to really make a difference to the environment	Adapted from Allison et al.[6], Bandura et al.[5] and Wakefield et al. [10]
	Priorities (2)	23. Often another household activity takes precedence over putting my food waste into a separate bin<br>24. I have too many things to think about other than whether or not I recycle my food waste	Adapted from West et al.[15] and Wakefield et al. [10]
	Attitude (4)	25. I think separating your food waste if a positive thing to do<br>26. I think it is important to recycle food waste<br>27. Recycling food waste is something I really care about<br>28. I find separating food waste to be an unpleasant task	Adapted from Wakefield et al. [10] Ajzen et al. [8]
	Positive reinforcement (2)	29. When I recycle my food waste, I feel like I'm making a difference<br>30. I feel good about myself when I recycle my food waste	Adapted from Skinner et al.[16] Wakefield et al. [10]
	Role/Identity (3)	31. It is my responsibility as a citizen to recycle my food waste<br>32. Recycling food waste is something people like me do<br>33. Recycling food waste is something that is important to me as a person	Adapted from Cane et al.[2] and Wakefield et al. [10]

References

1. O'Brien, J.; Thondhlana, G. Plastic bag use in South Africa: Perceptions, practices and potential intervention strategies. *Waste Management* **2019**, *84*, 320-328.
2. Cane, J.; O'Connor, D.; Michie, S. Validation of the theoretical domains framework for use in behaviour change and implementation research. *Implementation science* **2012**, *7*, 37.
3. Michie, S.; Van Stralen, M.M.; West, R. The behaviour change wheel: a new method for characterising and designing behaviour change interventions. *Implementation Science* **2011**, *6*, 42.
4. Taufik, D.; Reinders, M.J.; Molenveld, K.; Onwezen, M.C. The paradox between the environmental appeal of bio-based plastic packaging for consumers and their disposal behaviour. *Science of the Total Environment* **2020**, *705*, 135820.
5. Bandura, A. Health promotion from the perspective of social cognitive theory. *Psychology and health* **1998**, *13*, 623-649.
6. Allison, A.L.; Lorencatto, F.; Michie, S.; Miodownik, M. Influences on single-use and reusable cup use: a multidisciplinary mixed-methods approach to designing interventions reducing plastic waste. *UCL Open: Environment Preprint* **2020**.
7. Oliveira, V.; Sousa, V.; Vaz, J.; Dias-Ferreira, C. Model for the separate collection of packaging waste in Portuguese low-performing recycling regions. *Journal of environmental management* **2018**, *216*, 13-24.
8. Ajzen, I. The theory of planned behavior. *Organizational behavior and human decision processes* **1991**, *50*, 179-211.
9. Khan, F.; Ahmed, W.; Najmi, A.; Younus, M. Managing plastic waste disposal by assessing consumers' recycling behavior: the case of a densely populated developing country. *Environmental Science and Pollution Research* **2019**, *26*, 33054-33066.
10. Wakefield, A.; Axon, S. "I'ma bit of a waster": Identifying the enablers of, and barriers to, sustainable food waste practices. *Journal of Cleaner Production* **2020**, *275*, 122803.
11. Cialdini, R.B.; Kallgren, C.A.; Reno, R.R. A focus theory of normative conduct: A theoretical refinement and reevaluation of the role of norms in human behavior. In *Advances in experimental social psychology*, Elsevier: 1991; Vol. 24, pp. 201-234.
12. Russell, S.V.; Young, C.W.; Unsworth, K.L.; Robinson, C. Bringing habits and emotions into food waste behaviour. *Resources, Conservation and Recycling* **2017**, *125*, 107-114.
13. Verplanken, B.; Whitmarsh, L. Habit and climate change. *Current Opinion in Behavioral Sciences* **2021**, *42*, 42-46.
14. Sniehotta, F.F.; Schwarzer, R.; Scholz, U.; Schüz, B. Action planning and coping planning for long-term lifestyle change: theory and assessment. *European Journal of Social Psychology* **2005**, *35*, 565-576.
15. West, R.; Brown, J. Theory of addiction. **2013**.
16. Skinner, B.F. Operant behavior. *American psychologist* **1963**, *18*, 503.
