# Supplementary material for: Barriers and Enablers to Food Waste Recycling: A Mixed Methods Study amongst UK Citizens
Source: Int J Environ Res Public Health. 2022 Feb 26;19(5):2729. doi: 10.3390/ijerph19052729 (PMC8910430; doi:10.3390/ijerph19052729)
Supplement: Supplementary file 1 [file ijerph-19-02729-s001.zip › S2. Supplementary analyses.pdf]

**Supplementary File S2. Supplementary analyses**

**Source and location of food waste bins**

Shown in Table B1., the majority of participants who said yes to recycling their food waste via council services (n = 809) sourced their household food waste bin freely via their local council (n = 588). Those who indicated 'other' (n = 33) specified how they sourced their food waste bins. Some of these responses fell under one of the four predefined response categories or could not be coded and so were omitted (n = 21). Other responses included already having a food waste bin in the kitchen when they came to buy/rent their home (n = 8), not using a container at all and directly putting waste in the council bin (n = 2), having a separate compartment for food waste in an existing bin (n = 1) and acquiring one from a second-hand website (n = 1).

**Table B1. Table showing how participants sourced their food waste bins**

| How bin was sourced | Frequency (%) |
|---|---|
| Free from local council | 588 (72.7) |
| Purchased specifically for this purpose | 120 (14.8) |
| Repurposed another container | 63 (7.8) |
| Got given one by someone they know | 5 (0.6) |
| Other | 33 (4.1) |

As shown in Table B2, the majority of respondents keep their food waste bins on their kitchen countertops. Those who indicated other specified where they keep their bins. Those who indicated 'other' (n = 180) specified where in the home they keep their food waste bin. Some of these responses fell under one of the four predefined response categories or could

not be coded and so were omitted (n = 18). Of remaining 162 responses, most participants (n = 75) kept their household food waste caddies in some kind of outside area including sheds, just outside their back doors, gardens or balcony/porches. Windowsills (n = 17), garages (n = 13) and utility rooms (n = 25) were other reported locations. Participants also kept food waste in their fridge/freezers (n = 11). Six participants reported keeping food waste bins on the floor. Other areas included in the utility/kitchen sink (n = 6), in the conservatory (n = 2), a cupboard in another area of the house (n = 2), under the kitchen table (n = 1), on the kitchen fridge (n = 1), hung to the back of a door (n = 1), the kitchen draining board (n = 1) and the dining room (n = 1).

**Table B2. Table showing where participants store their food waste bin**

| Where bin is located in home | Frequency (%) |
|---|---|
| On the kitchen countertop | 448 (55.4) |
| Under the sink | 125 (15.6) |
| In a kitchen cupboard | 30 (3.7) |
| Integrated pull out bin | 26 (3.2) |
| Other | 180 (22.2) |
